# Supplementary material for: Assessing large language model performance related to aging in genetic conditions
Source: NPJ Aging. 2025 May 3;11(1):33. doi: 10.1038/s41514-025-00226-z (PMC12049513; doi:10.1038/s41514-025-00226-z)
Supplement: Supplementary file 1 — Supplementary Figures and Tables [file 41514_2025_226_MOESM1_ESM.pdf]

Supplementary Table 1. Scoring rubric for the medical vignettes and management plans. We assessed all vignette (n=282) and management responses (n=53) for Correctness, Completeness, and Conciseness, assigning a score of either 0 or 1 for each metric. For the medical vignettes, since all results received Conciseness score of 1, we removed this metric when evaluating the Total score of the vignettes, thus allowing a maximum Total score of 2 for each vignette. For management results, Conciseness was still included in the analysis, yielding a maximum Total score of 3 for each management answer.

**Criteria**	**Score**	**Definition**
**Correctness***	0	Inaccurate and inappropriate information or description of manifestations not relevant to the genetic disorder, the patient's age, or sex.
	1	Accurate and appropriate description of manifestations (presented without errors) of the genetic disorder considering the patient's age and sex.
**Completeness***	0	Missing crucial patient information or cardinal clinical or laboratory features of the genetic disorder.
	1	Provides a complete list of important clinical or laboratory features relevant to the genetic disorder.**
**Conciseness**	0	Includes unnecessary or repetitive information or lacks focus on key points.
	1	Clearly and concisely presents relevant information without unnecessary or repetitive details.

***** A separate metric "Accuracy" was marked as 1 only if a vignette received a score of 1 for both correctness and completeness.

** Considering that not all individuals affected by a genetic condition display all the textbook characteristics, but instead demonstrate varying degrees of severity, clinicians evaluating the vignettes considered a vignette complete if it exhibited the most essential features along with sufficient additional clinical and laboratory indicators for a given condition.

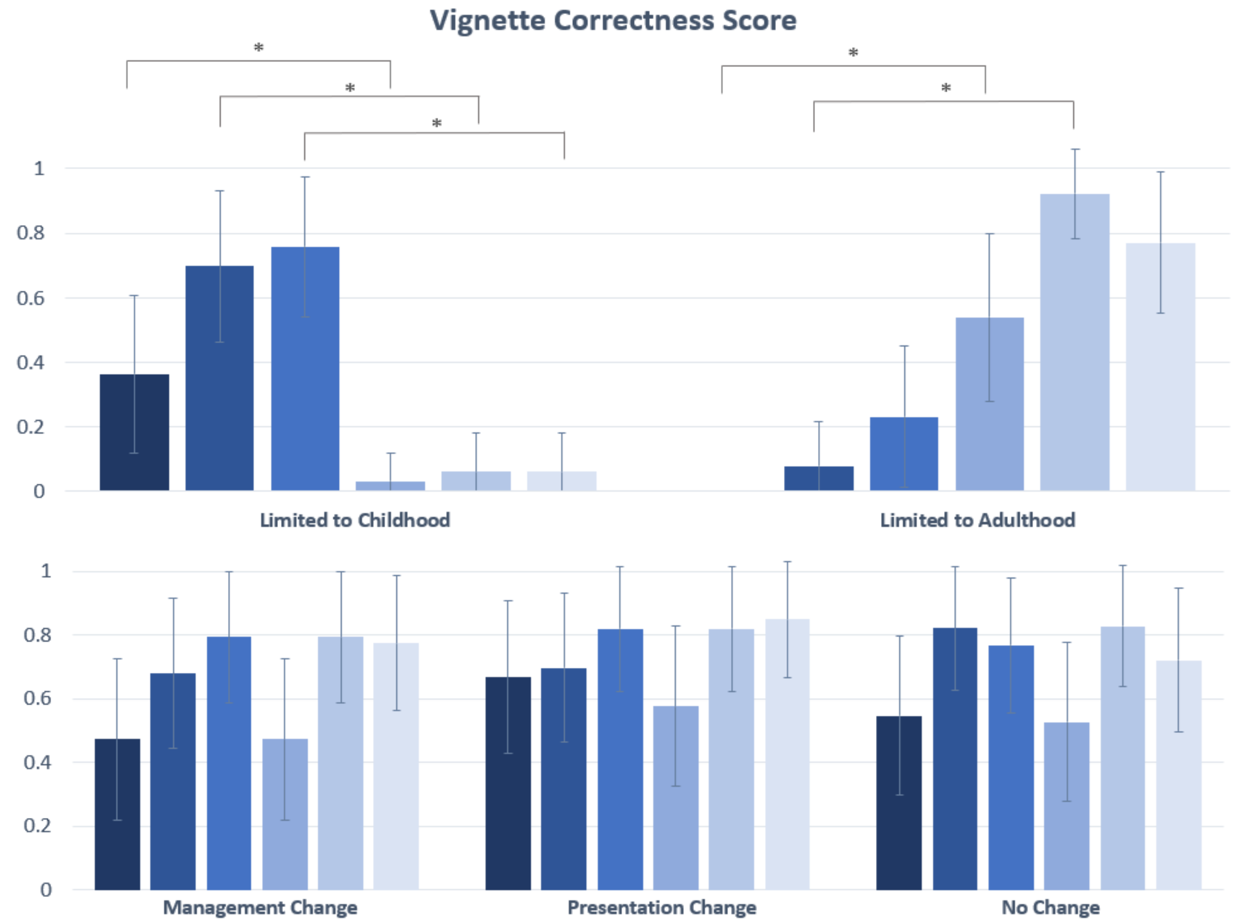

Supplementary Figure 1. Individual Correctness score breakdown for medical vignettes with error and significance in all 5 categories (Limited to Childhood, Limited to Adulthood, Management Change, Presentation Change, and No Change). All vignettes were clinician-graded and assigned a score of either 0 or 1 for Correctness. One asterisk (*) indicates significance between child and adult vignettes for the Correctness score. In this chart, significance is only demonstrated for comparisons between child and adult vignettes for the same model (see Supplementary Tables 7 and 8 for statistical comparisons between 70b, 70b Context and GPT).

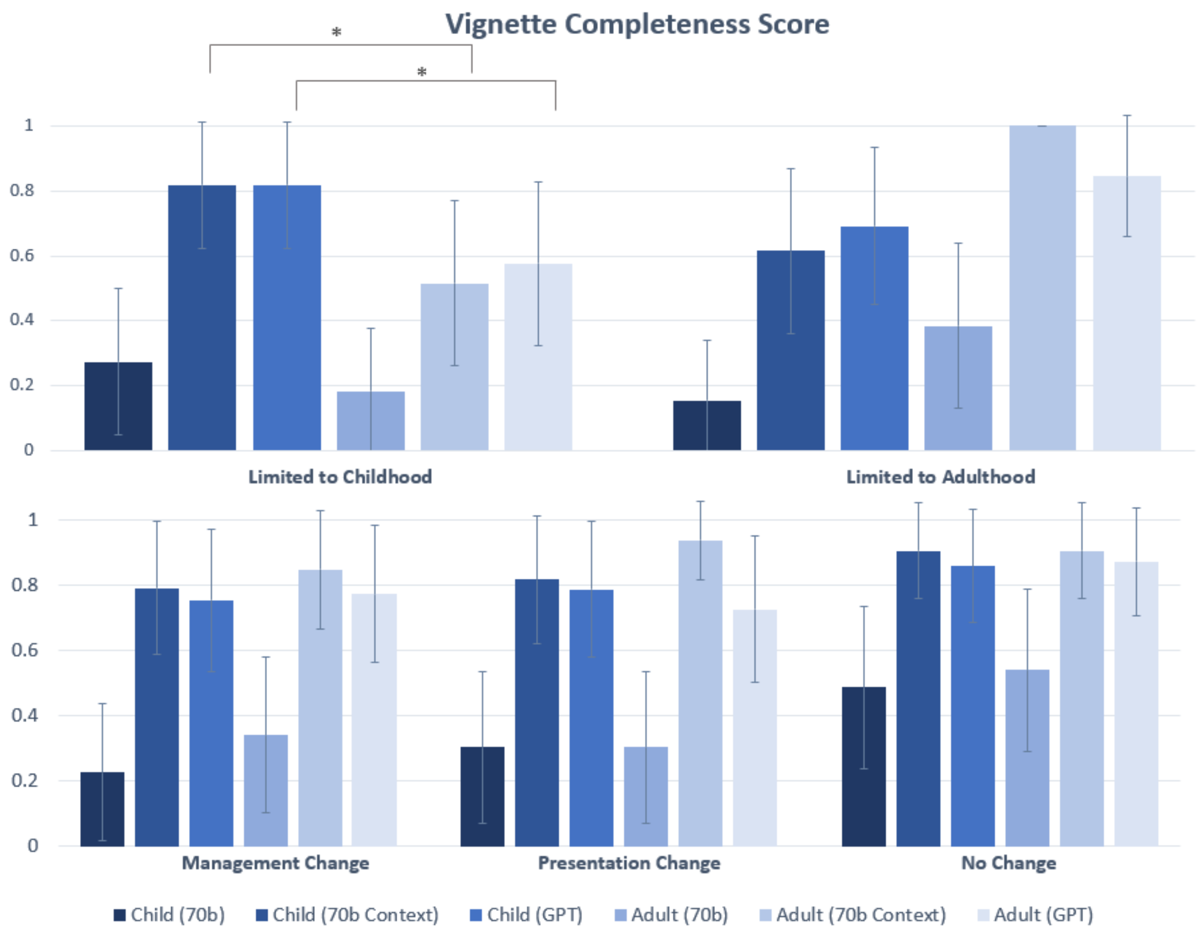

Supplementary Figure 2. Individual Completeness score breakdown for medical vignettes with error and significance for all 5 categories (Limited to Childhood, Limited to Adulthood, Management Change, Presentation Change, and No Change). All vignettes were clinician-graded and assigned a score of either 0 or 1 for Completeness. One asterisk (*) indicates significance between child and adult vignettes for the Completeness score. In this chart, significance is only demonstrated for comparisons between child and adult vignettes for the same model (see Supplementary Tables 7 and 8 for statistical comparisons between 70b, 70b Context and GPT).

Supplementary Table 2. Statistical comparison of llama-2-70b-chat vignette scores with and without in-context prompting (70b vs 70b Context). Paired t-tests were conducted between the Correctness, Completeness, and Accuracy scores of clinician-graded medical vignettes.

Category	Correctness (p-value)		Completeness (p-value)		Accuracy (p-value)	
	Child	Adult	Child	Adult	Child	Adult
Limited to Childhood	0.00296	0.325	1.58×10^{-5}*	0.00296	0.00300	0.325
Limited to Adulthood	0.337	0.0180	0.00753	0.000893*	0.337	0.00281
Management Change	0.0329	0.000371*	4.28×10^{-10}*	3.86×10^{-8}*	2.99×10^{-5}*	2.15×10^{-7}*
Presentation Change	0.744	0.0184	1.02×10^{-5}*	1.61×10^{-8}*	0.00123*	1.77×10^{-6}*
No Change	2.23×10^{-8}*	1.40×10^{-11}*	8.66×10^{-17}*	7.76×10^{-13}*	3.41×10^{-15}*	6.12×10^{-17}*

*The Bonferroni threshold for significance is p<0.00167.

Supplementary Table 3. Statistical comparison of llama-2-70b-chat vignette scores with in-context prompting to GPT-3.5 vignette scores (70b Context vs GPT). Paired t-tests were conducted between the Correctness, Completeness, and Accuracy scores of clinician-graded medical vignettes. None of these values are significant at the $\alpha = 0.05$ significance level using the Bonferroni correction ($p < 0.00167$).

Category	Correctness (p-value)		Completeness (p-value)		Accuracy (p-value)	
	Child	Adult	Child	Adult	Child	Adult
Limited to Childhood	0.587	1	1	0.627	0.436	1
Limited to Adulthood	0.298	0.298	0.695	0.165	0.298	0.150
Management Change	0.190	0.816	0.646	0.325	0.319	0.151
Presentation Change	0.257	0.746	0.761	0.0215	0.608	0.179
No Change	0.255	0.0274	0.209	0.358	0.126	0.0117

*The Bonferroni threshold for significance is p<0.00167.

Supplementary Table 4. Potential cross-category conditions (PCCs) that were removed from the original categories “Management Change”, “Presentation Change”, and “No Change” for sub analysis on vignette scores. PCCs are conditions that may potentially fall into multiple categories, based on variability of presentation and management across the lifespan. No conditions in the “Limited to childhood” and “Limited to adulthood” categories qualified as PCCs, and thus were not removed.

**Potential cross-category conditions (n= 23)**		
Autosomal dominant polycystic kidney disease	Glycogen storage disease due to acid maltase deficiency	Metachromatic leukodystrophy
Biotinidase deficiency	GM1 gangliosidosis	Niemann-Pick disease type C
Carnitine palmitoyltransferase II deficiency	Homocystinuria due to cystathionine beta-synthase deficiency	Ornithine transcarbamylase deficiency
Chronic visceral acid sphingomyelinase deficiency	Huntington disease	Propionic acidemia
Cystinosis	Krabbe disease	Proximal spinal muscular atrophy
Fabry disease	Laminin subunit alpha 2-related congenital muscular dystrophy	Sandhoff disease
Gaucher disease	Lysosomal acid lipase deficiency	Steinert myotonic dystrophy
Tay-Sachs disease	Trichothiodystrophy	

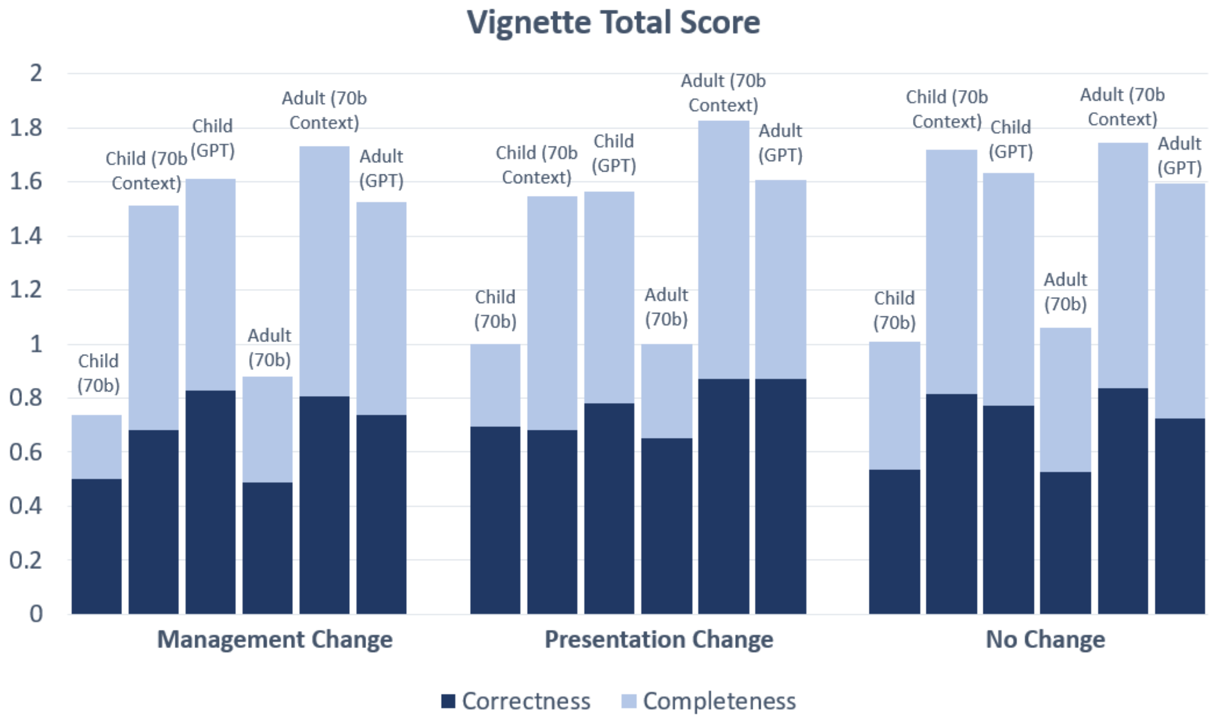

Supplementary Figure 3. Correctness and Completeness score breakdowns for vignettes in "Management Change", "Presentation Change", and "No Change" categories, after removing all potential cross-category conditions (PCCs). PCCs are listed in Supplementary Table 4. All vignettes were clinician-graded and assigned a score of either 0 or 1 for Correctness and Completeness, for a total of 2 possible points. Significance (*) is indicated at the $\alpha = 0.05$ level between child and adult vignettes for the Correctness score only. Two markers (**) indicates significance at the $\alpha = 0.05$ level between child and adult scores for both the Correctness and Completeness scores. In this chart, significance is only demonstrated for comparisons between child and adult vignettes for the same model (See Supplementary Tables 9 and 10 for statistical comparisons between 70b, 70b Context and GPT). The Bonferroni threshold for significance is $p < 0.005$. This chart shows the combined Correctness and Completeness scores; See Supplementary Figures 5 and 6 for individual Correctness and Completeness graphs with variance.

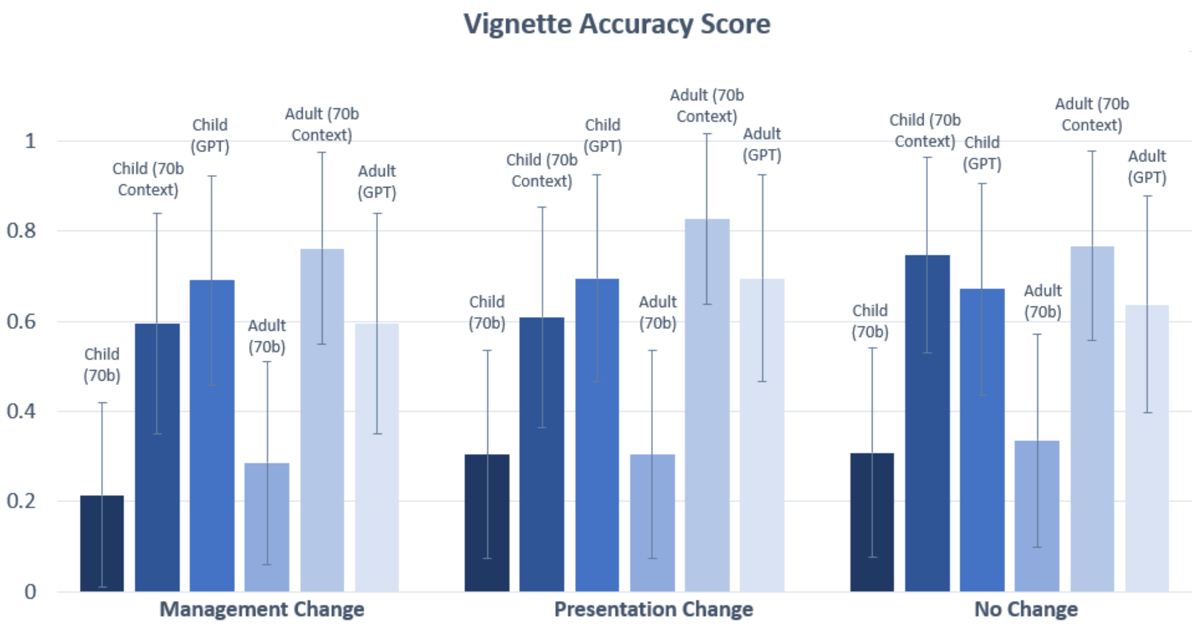

Supplementary Figure 4. Vignette Accuracy score for vignettes in "Management Change", "Presentation Change", and "No Change" categories, after removing all potential cross-category conditions (PCCs). PCCs are listed in Supplementary Table 4. Statistically significant differences (*) in Accuracy scores are indicated at the $\alpha = 0.05$ level between child and adult vignettes. In this chart, significant differences are only observed for comparisons between child and adult vignettes for the same model (see Supplementary Tables 9 and 10 for statistical comparisons between 70b, 70b Context and GPT). The Bonferroni threshold for significance is $p < 0.005$.

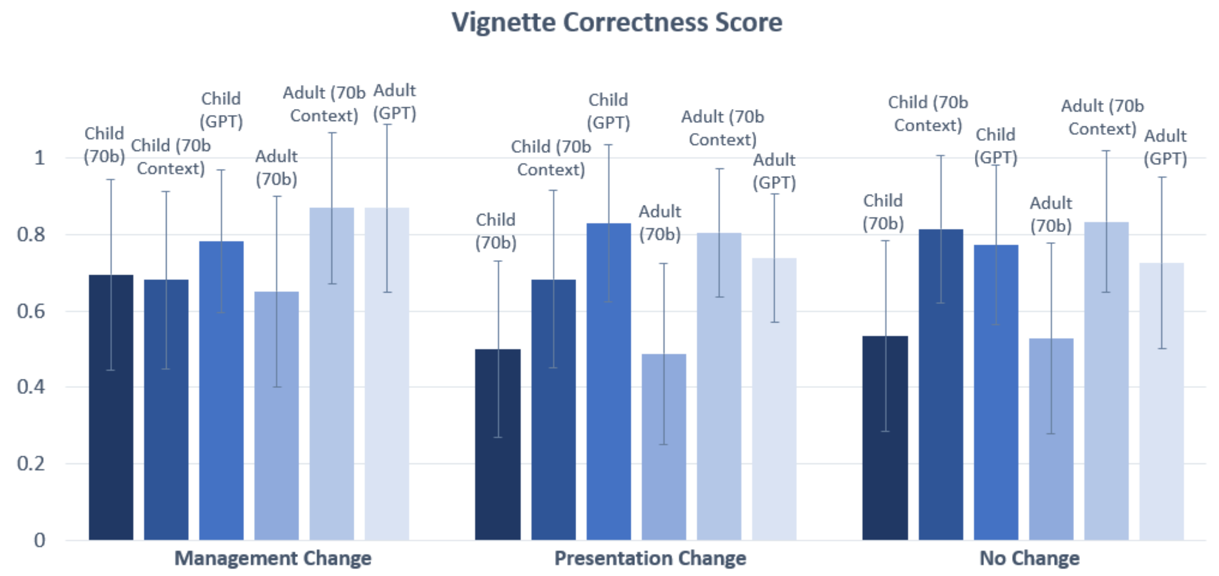

Supplementary Figure 5. Individual Correctness score breakdown for medical vignettes with error and significance in "Management Change", "Presentation Change", and "No Change" categories, after removing all potential cross-category conditions (PCCs). PCCs are listed in Supplementary Table 4. All vignettes were clinician-graded and assigned a score of either 0 or 1 for Correctness. One asterisk (*) indicates significance between child and adult vignettes for the Correctness score. In this chart, significance is only demonstrated for comparisons between child and adult vignettes for the same model (see Supplementary Tables 9 and 10 for statistical comparisons between 70b, 70b Context and GPT).

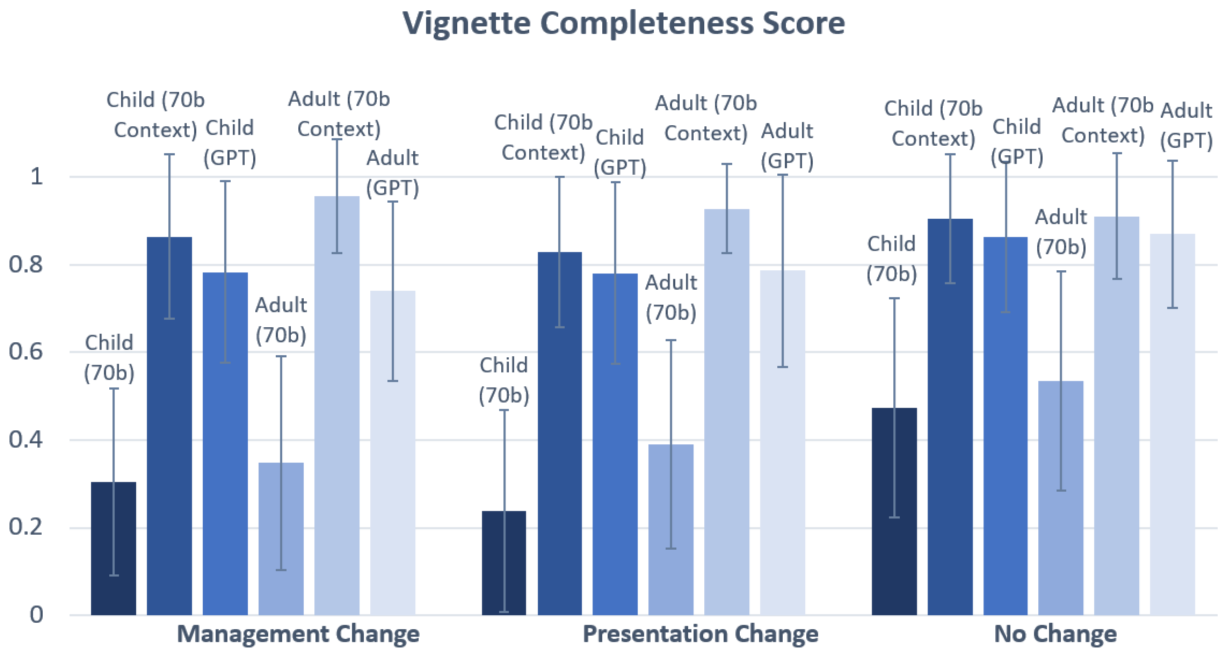

Supplementary Figure 6. Individual Completeness score breakdown for medical vignettes with error and significance in "Management Change", "Presentation Change", and "No Change" categories, after removing all potential cross-category conditions (PCCs). PCCs are listed in Supplementary Table 4. All vignettes were clinician-graded and assigned a score of either 0 or 1 for Completeness. One asterisk (*) indicates significance between child and adult vignettes for the Completeness score. In this chart, significance is only demonstrated for comparisons between child and adult vignettes for the same model (see Supplementary Tables 9 and 10 for statistical comparisons between 70b, 70b Context and GPT).

Supplementary Table 5. Statistical comparison of llama-2-70b-chat vignette scores with and without in-context prompting (70b vs 70b Context), for “Management change”, “Presentation change”, and “No change” categories, after removing all potential cross-category conditions (PCCs). PCCs are listed in Supplementary Table 4. Paired t-tests were conducted between the Correctness, Completeness, and Accuracy scores of clinician-graded medical vignettes.

Category	Correctness (p-value)		Completeness (p-value)		Accuracy (p-value)	
	Child	Adult	Child	Adult	Child	Adult
Management Change	0.109	0.00333	3.54×10^{-8}*	1.11×10^{-6}*	2.88×10^{-4}*	6.00×10^{-6}*
Presentation Change	0.664	0.0568	3.84×10^{-4}*	8.90×10^{-6}*	0.0161	7.74×10^{-5}*
No Change	2.15×10^{-8}*	2.80×10^{-12}*	7.17×10^{-17}*	2.73×10^{-13}*	2.94×10^{-15}*	1.42×10^{-17}*

*The Bonferroni threshold for significance is p<0.00167.

Supplementary Table 6. Statistical comparison of llama-2-70b-chat vignette scores with in-context prompting to GPT-3.5 vignette scores (70b Context vs GPT), for “Management change”, “Presentation change”, and “No change” categories, after removing all potential cross-category conditions (PCCs). PCCs are listed in Supplementary Table 4. Paired t-tests were conducted between the Correctness, Completeness, and Accuracy scores of clinician-graded medical vignettes. None of these values are significant at the $\alpha = 0.05$ significance level using the Bonferroni correction ($p < 0.00278$).

Category	Correctness (p-value)		Completeness (p-value)		Accuracy (p-value)	
	Child	Adult	Child	Adult	Child	Adult
Management Change	0.0510	0.623	0.486	0.133	0.352	0.0701
Presentation Change	0.185	1	0.575	0.0218	0.328	0.266
No Change	0.388	0.00482	0.290	0.258	0.190	0.00243

*The Bonferroni threshold for significance is p<0.00167.

Supplementary Table 7. Sex bias in LLM vignette output according to LLM and prompt type. Of the 282 conditions in this study, 34 are known to preferentially affect males and 12 are known to preferentially affect females (based on source data from Orphanet: https://www.orpha.net/en/disease). However, removing conditions with a known male/female predilection did not ameliorate the sex bias produced by the LLMs. Sex output was more equitable in the adult prompted vignettes compared to the child for both LLM types. Interestingly, when context was provided to the 70b prompt, sex ratio screwed more heavily female for adult vignettes. Of note, GPT did not provide sex identifiers for 23 (8.1%) of the child vignettes, whereas in the adult prompt it did not provide sex identifiers for just 2 vignettes. Sex identifiers were given in 70b generated vignettes except for 1 in Adult (70b) and Child (70b Context).

	**All Conditions (n=282)**			**Conditions that affect males and females proportionately (n=236)**		
	Male (M)	Female (F)	M:F Ratio	Male (M)	Female (F)	M:F Ratio
**Child (70b)**	248	34	7.3:1	210	26	8.1:1
**Child (70b Context)**	234	47	5:1*	199	36	5.5:1*
**Child (GPT)**	211	48	4.4:1*	174	41	4.2:1*
**Adult (70b)**	167	114	1.5:1*	134	101	1.3:1*
**Adult (70b Context)**	100	82	1:1.8	72	164	1:2.3
**Adult (GPT)**	205	75	2.7:1*	171	64	2.7:1

*Sex or sex identifiers were not given for all vignettes.

Supplementary Table 8. Conditions in this study that disproportionately affect males or females. The Orphanet database (https://www.orpha.net/en/disease) served as the source of information about sex specific prevalence unless otherwise noted by the superscript letters and citations below.

**Conditions that disproportionately affect Males (n=34)**		**Conditions that disproportionately affect Females (n=12)**
Aarskog-Scott syndrome	Mucopolysaccharidosis type 2	Acute intermittent porphyria
Barth syndrome	Oculocerebrorenal syndrome of Lowe	Crouzon syndrome-acanthosis nigricans syndrome
Brugada syndrome	Ornithine transcarbamylase deficiency	Currarino syndrome
Chronic granulomatous disease	Paroxysmal non-kinesigenic dyskinesia	Incontinentia pigmenti
Complete androgen insensitivity syndrome	Pelizaeus-Merzbacher disease	Orofaciodigital syndrome type 1
Duchenne muscular dystrophy	Recessive X-linked ichthyosis	Porphyria variegate
Fabry disease	Retinitis pigmentosa	Pseudoxanthoma elasticum
Fragile X syndrome	Shwachman-Diamond syndrome[b]	Rett syndrome
Glycogen storage disease due to liver phosphorylase kinase deficiency[a]	Syndromic recessive X-linked ichthyosis	Trisomy 18
Hemophilia A	Triploidy[c]	Turner syndrome
Hemophilia B	Wiskott-Aldrich syndrome	Wolf-Hirschhorn syndrome
Hypohidrotic ectodermal dysplasia	X-linked adrenal hypoplasia congenita	X-linked dominant chondrodysplasia punctata
Kallmann syndrome	X-linked agammaglobulinemia	
Leber hereditary optic neuropathy	X-linked centronuclear myopathy	
Leigh syndrome	X-linked Emery-Dreifuss muscular dystrophy	
Lesch-Nyhan syndrome	X-linked recessive ocular albinism	
Menkes disease	X-linked retinoschisis	

a. Kishnani PS, Goldstein J, Austin SL, Arn P, Bachrach B, Bali DS, Chung WK, El-Gharbawy A, Brown LM, Kahler S, Pendyal S, Ross KM, Tsilianidis L, Weinstein DA, Watson MS; ACMG Work Group on Diagnosis and Management of Glycogen Storage Diseases Type VI and IX. Diagnosis and management of glycogen storage diseases type VI and IX: a clinical practice resource of the American College of Medical Genetics and Genomics (ACMG). Genet Med. 2019 Apr;21(4):772-789. Doi: 10.1038/s41436-018-0364-2. Epub 2019 Jan 19. PMID: 30659246.

b. Farooqui SM, Ward R, Aziz M. Shwachman-Diamond Syndrome. [Updated 2023 Jul 17]. In: StatPearls [Internet]. Treasure Island (FL): StatPearls Publishing; 2024 Jan-. Available from:

Kolarski M, Ahmetovic B, Beres M, Topic R, Nikic V, Kavecan I, Sabic S. Genetic Counseling and Prenatal Diagnosis of Triploidy During the Second Trimester of Pregnancy. Med Arch. 2017 Apr;71(2):144-147. Doi: 10.5455/medarh.2017.71.144-147. PMID: 28790549; PMCID: PMC5511524.

Supplementary Table 9. Vignette sex output for conditions that disproportionately affect males or females. Of the 282 conditions in this study, 34 are known to preferentially affect males and 12 are known to preferentially affect females. The majority of the male conditions (22/34) can affect females, some rarely (e.g., Wiskott-Aldrich syndrome) and some with a more minor disproportionate ratio (e.g., Paryoxysmal non-kinesigenic dyskinesia, 1.4 males affected for each female). For females, only 2 out of the 12 conditions are known to exclusively affect females (Rett and Turner syndrome), whereas the other conditions vary in sex ratios.

	**Conditions that disproportionately affect Males (n=34)**			**Conditions that disproportionately affect Females (n=12)**		
	Male (M)	Female (F)	M:F Ratio	Male (M)	Female (F)	M:F Ratio
**Child (70b)**	32	2	16:1	6	6	1:1
**Child (70b Context)**	31	3	10.3:1	4	8	1:2
**Child (Chat GPT)**	33	1	33:1	4	6	1:1.5*
**Adult (70b)**	32	2	16:1	1	11	1:11
**Adult (70b Context)**	27	7	3.9:1	1	11	1:11
**Adult (Chat GPT)**	32	1	32:1	2	10	1:5

*Sex or sex identifiers were not provided for 2 vignettes.

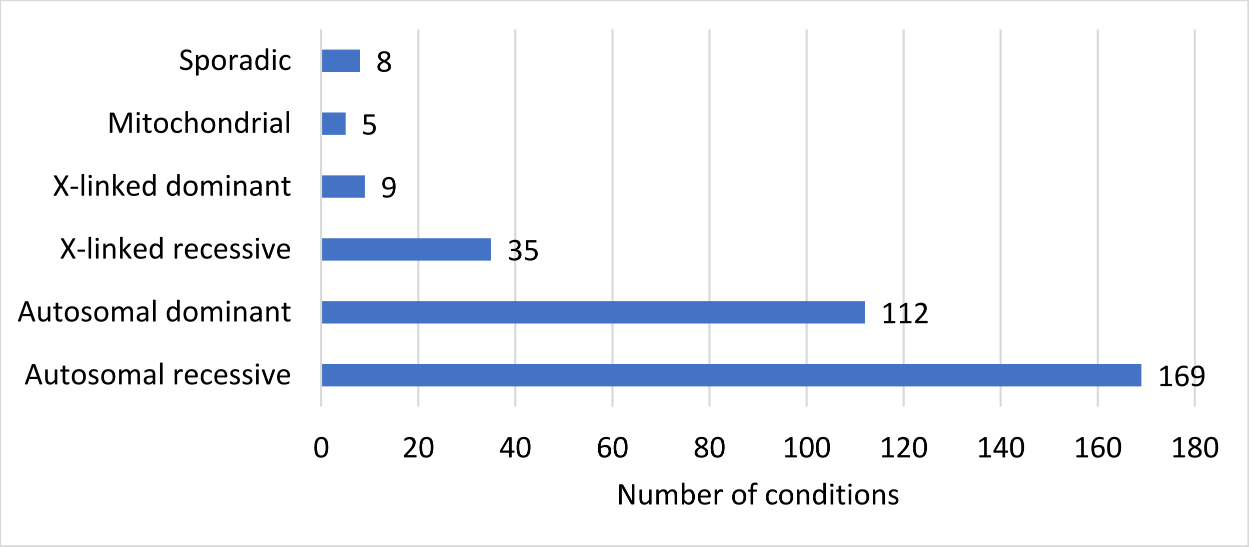

Supplementary Figure 7. Mode of inheritance for the 282 conditions included in this study. Forty-five of these conditions have multiple modes of inheritance. Therefore, a single condition could represent multiple points. In addition to hereditary conditions, there are 8 conditions that are typically sporadic.

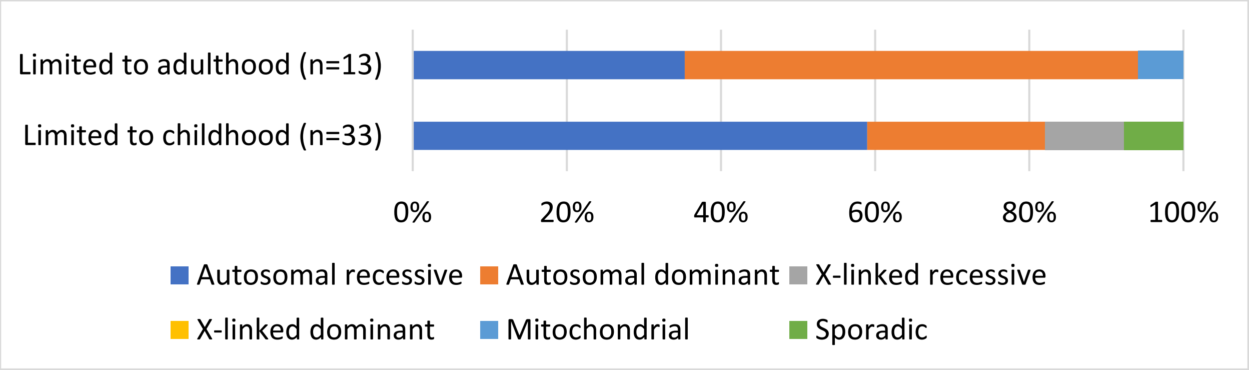

Supplementary Figure 8. Distribution of modes of inheritances for "Limited to Childhood" and "Limited to Adulthood" conditions. While autosomal recessive conditions are predominant in "Limited in Childhood" conditions, autosomal dominant conditions are more common in "Limited to Adulthood" conditions.

Supplementary Table 10. For the following 6 diseases, Roberta embedding was used to compute the cosine similarity score between each of the 10 repeated vignette generations with the original vignette. Similarity score ranges from -1 to 1, with 1 as the most similar. In the last row, one additional comparison was made (Tibial Muscular Dystrophy- child vs Fryns syndrome- adult) to demonstrate what the similarity scores are for two dissimilar vignettes. To further provide context about these similarity scores, we also computed the score between two highly similar sentences: "I like ice cream" and "I love ice cream" and found the similarity score to be 0.891.

	1	2	3	4	5	6	7	8	9	10	Average
Tibial Muscular Dystrophy (child)	0.712	0.865	0.633	0.914	0.636	0.564	0.691	0.948	0.711	0.780	0.745
Tibial Muscular Dystrophy (adult)	0.912	0.943	0.921	0.898	0.909	0.941	0.934	0.919	0.950	0.927	0.925
Amyotrophic lateral sclerosis (child)	0.789	0.843	0.811	0.803	0.774	0.791	0.613	0.789	0.807	0.648	0.767
Amyotrophic lateral sclerosis (adult)	0.860	0.908	0.799	0.650	0.884	0.909	0.855	0.871	0.881	0.841	0.846
Leber hereditary optic neuropathy (child)	0.804	0.744	0.744	0.781	0.753	0.640	0.754	0.750	0.718	0.796	0.748
Leber hereditary optic neuropathy (adult)	1.000	1.000	0.831	0.898	0.920	0.894	0.905	0.932	0.929	0.862	0.917
Aicardi-Goutières syndrome (child)	0.794	0.794	0.850	0.901	0.932	0.935	0.898	0.898	0.915	0.930	0.885
Aicardi-Goutières syndrome (adult)	0.951	0.836	0.906	0.808	0.832	0.922	0.914	0.928	0.918	0.940	0.896
Fryns syndrome (child)	0.802	0.711	0.887	0.830	0.672	0.797	0.832	0.718	0.752	0.851	0.785
Fryns syndrome (adult)	0.912	0.844	0.833	0.882	0.868	0.900	0.891	0.851	0.918	0.828	0.873
Meckel syndrome (child)	0.782	0.812	0.683	0.782	0.717	0.814	0.672	0.746	0.835	0.821	0.767
Meckel syndrome (adult)	0.706	0.804	0.733	0.746	0.715	0.721	0.756	0.747	0.808	0.752	0.749
Tibial Muscular Dystrophy (child) vs Fryns (adult)	0.407	0.415	0.484	0.446	0.401	0.417	0.609	0.413	0.440	0.509	0.454

Supplementary Table 11. Conditions chosen for the vignettes to be scored by two clinicians for evaluation of agreement on Correctness and Completeness scores by the Kappa statistic. Results of the clinician agreement Kappa statistic in Supplementary Table 10.

Condition Name	Category	Condition Name	Category
Holocarboxylase synthetase deficiency	Limited to Childhood	Huntington disease	Presentation Change
Werner syndrome	Limited to Adulthood	Osteogenesis imperfecta	Presentation Change
Birt-Hogg-Dubé syndrome	Limited to Adulthood	Angelman syndrome	Presentation Change
22q11.2 deletion syndrome	Management Change	Sotos syndrome	Presentation Change
Neurofibromatosis type 1	Management Change	Fabry disease	Presentation Change
Alpha-1-antitrypsin deficiency	Management Change	Hereditary fructose intolerance	Presentation Change
Cystic fibrosis	Management Change	Multiple endocrine neoplasia type 1	Presentation Change
Duchenne muscular dystrophy	Management Change	Pseudoachondroplasia	No Change
Wilson disease	Management Change	Kabuki syndrome	No Change
Turner syndrome	Management Change	Hypochondroplasia	No Change
Prader-Willi syndrome	Management Change	Hemophilia B	No Change
Syndromic recessive X-linked ichthyosis	Management Change	Leigh syndrome	No Change
Axenfeld-Rieger syndrome	Management Change	3-methylcrotonyl-CoA carboxylase deficiency	No Change
Stickler syndrome	Presentation Change	Oculocutaneous albinism type 2	No Change
Medium chain acyl-CoA dehydrogenase deficiency	Presentation Change	Diastrophic dysplasia	No Change

Supplementary Table 12. Kappa statistic to evaluate the agreement among the clinicians while grading the vignettes for Correctness and Completeness. Kappa statistic close to 1 indicates the highest agreement, whereas 0 indicates no agreement.

	Clinician 1 & Clinician 2	Clinician 1 & Clinician 3	Clinician 2 & Clinician 3
Kappa statistic (Correctness)	0.6	0.61	0.74
Kappa statistic (Completeness)	0.52	0.55	0.88

Supplementary Table 13. Scoring rubric for patient-geneticist dialogues on 5-point Likert scale. Since a dialogue is longer than a vignette and management plan, each of the three metrics was graded on a more comprehensive 5-point Likert scale to more precisely assess differences. Compassion metric was added to the patient-geneticist dialogue rubric to further evaluate the quality of communication provided by the LLMs.

**Criteria**	**Definition**	**1 (Strongly Disagree)**	**2 (Disagree)**	**3 (Uncertain)**	**4 (Agree)**	**5 (Strongly Agree)**
**Correctness**	The dialogue contains **realistic**, accurate and appropriate descriptions (or management) relevant to the genetic disorder, the patient's age, or sex.	Contains numerous errors, significantly impacting understanding.	Contains several errors, affecting understanding.	Contains occasional errors, slightly affecting understanding	Contains few errors but generally does not affect understanding of key concepts.	Contains no errors, greatly enhanced understanding.
**Completeness**	The dialogue provides a complete list of important clinical, laboratory features [HPO terms], and management options relevant to the genetic disorder	The dialogue lacks crucial information, leaving significant gaps in understanding and treatment planning.	The dialogue is missing some important details, leading to gaps in understanding and treatment planning.	The dialogue includes most relevant information but may lack some minor details.	The dialogue includes nearly all relevant information, facilitating comprehensive understanding and treatment planning.	The dialogue is very thorough, providing all necessary information for comprehensive understanding and treatment planning.
**Compassion**	The dialogue shows empathy, addresses concerns, provides reassurance, asks open-ended questions, provides resources, avoids jargon, and checks understanding.	Strongly Disagree	Disagree	Neither agree nor disagree	Agree	Strongly Agree
